# The Impact of the COVID-19 Pandemic on Families: Young People’s Experiences in Estonia

**DOI:** 10.3389/fsoc.2021.732984

**Published:** 2021-08-25

**Authors:** Dagmar Kutsar, Leena Kurvet-Käosaar

**Affiliations:** ^1^ Institute of Social Studies, University of Tartu, Tartu, Estonia; ^2^ Institute of Cultural Research, University of Tartu, Tartu, Estonia

**Keywords:** children’s perspectives on COVID-19, distance learning, family dynamics, impact of lockdown, social contacts, young people’s subjective well-being

## Abstract

This articles reflects the impacts of the COVID-19 pandemic on the everyday lives of children and their families in Estonia during lockdown in spring 2020 and 2021. The data corpus is based on diaries compiled by children during the first lockdown in 2020 for a collection at the Estonian Literary Museum, and on a series of semi-structured interviews with children documenting their experiences during lockdown in spring 2021. The study draws on literature from the “new sociology of childhood” and applies Bronfenbrenner’s social ecological model to an analysis of young people’s experiences when their mobility outside the home was restricted, and they were forced to reorganise their time use. The findings show how the pandemic extended the social contexts in which children and their families are embedded and highlighted the role played by socio-cultural factors in shaping children’s coping capacities. In combination, analysis of the two datasets demonstrated the differential effects of lockdown on young children. The accounts from the first wave of the pandemic in 2020 suggested that positive family environments could smooth the negative effects of lockdown and help them cope with unexpected changes in their everyday lives. The interviews during the second outbreak of the pandemic revealed how the emerging weariness and boredom reported by some children strained family relationships. The amount of time that children spent online both modified and expanded their experiences of technology-supported interactive spaces. Their reports showed that the interactive contexts in which they were operating through social media extended beyond national borders to an interest in transnational and global events. Online communication did not, however, compensate for the loss of real-life contacts with friends, which became a major concern for young people in Estonia. In the concluding discussion, the authors consider policy responses that address the main issues identified in the research.

## Introduction

The World Health Organisation declared a state of international public health emergency on 30 January 2020 and a global pandemic on 11 March 2020. Lockdown was promptly proclaimed in the Baltic States: in Estonia and Latvia on 12 March 2020, and in Lithuania four days later. From an economic perspective, the situation of the three Baltic States differed to a certain extent. Before the outbreak of the pandemic, Lithuania had experienced steady economic growth, while growth in Estonia and Latvia was slowing down and went into decline in the first quarter of 2020. The second quarter of 2020 was difficult in all three Baltic States, but the decline in GDP remained well below the EU27 average of −14%: it was lowest in Estonia (−4.6%) and highest in Latvia (−8.9%). A similar trend occurred in the unemployment rate: it increased least in Estonia and most in Latvia, while remaining below the EU average (Parliament of Estonia Foresight Centre, 2020b). The Bank of Latvia (Parliament of Estonia Foresight Centre, 2020a) noted that during the first wave of the pandemic, Estonia had disbursed more in support measures than Latvia and Lithuania, and furlough support was also more generous: 0.9% of GDP compared to 0.8% in Lithuania and 0.3% in Latvia.

In mid-October 2020, Estonia, Latvia and Lithuania were among the EU member states with the lowest COVID-19 infection and death rates in the EU. Estonia also reported the lowest numbers among the Baltic States (Our World in Data, 2021). Like most other Central and Eastern European countries, all three Baltic States had been relatively untouched by the first wave of the pandemic in March−April 2020. The second outbreak started in late October 2020 and peaked on 25 December 2020 in Lithuania and on 17 March 2021 in Estonia. Rates in Latvia fluctuated between the two dates. By June 2021, the number of confirmed new cases per million inhabitants had fallen to 130 in Latvia, 119 in Lithuania and 68 in Estonia; the deaths rates (rolling 7-days average) had fallen to 3.3 for both Latvia and Lithuania and 1.4 for Estonia.

The COVID-19 pandemic not only halted economic growth, it also constituted a huge challenge to health care capacity. Until the onslaught of the pandemic, Baltic societies had not experienced any major public health problems in recent years. As in other Western countries, the pandemic gave a new prominence to the role of scientists in informing political decisions and the ways in which government policies were communicated to the public.

The first lockdown was proclaimed on 12 March 2020 and was due to last until 1 May 2020 but was then extended to 17 May 2020. The second lockdown was in force from 11 March 2021 to 25 May 2021. Lockdown during the two waves of the pandemic involved substantively the same measures, although they were applied with different degrees of stringency and were not perceived as having the same impacts on the everyday lives of young people. During both waves schools switched to distance learning. Shopping centres, theatres, cinemas and other places of entertainment were closed. Social distancing was compulsory in public places. Wearing masks was recommended in 2020 and compulsory in 2021. The 2 + 2 rule was introduced, allowing meetings of two people from two different households out-of-doors. Government rules did not apply to the private sphere. Although private gatherings were not recommended, no restrictions were placed on meetings between friends. Nor were children of separated parents prevented from moving between households. Parents who commuted across borders for work were hit by border closures and by restrictions introduced in neighbouring countries.

During the lockdown in spring 2020, the government’s intention was to limit the impact of the pandemic on public health facilities by keeping society as closed as possible. Evidence of the harm caused by lockdown restrictions to economic and social life, in combination with greater knowledge about the virus, the introduction of effective vaccines and the adaptative capacity of the health services, persuaded the government to apply a lower level of stringency in 2021.

Drawing on data collected over more than a year of living with the COVID-19 pandemic, the authors of this article reflect on how children in families in Estonia coped with the severe disruptions it caused to their everyday lives. Estonia was selected for an exploratory study because it managed the pandemic relatively well, compared to many other EU member states. Estonia also stands out as a country that had benefitted fully from technological developments in the post-Soviet era. In a small economy with good recovery capacity, the IT sector proved to be an important factor in the successful management of everyday life during periods of severe restrictions on mobility and social interaction. IT-based services for schoolwork and online study, as well as other e-services such as digital medical prescriptions, were already an integral part of everyday life. Estonians were technically relatively well prepared to study and work from home.

During the first lockdown in spring 2020, the Estonian research team carried out new empirical work based on a corpus of diaries compiled by young people aged 12–13 and 17–18. The diaries were the result of a public data collection campaign organised by the Estonian Literary Museum. A year later in spring 2021, the research team at Tartu University conducted a series of semi-structured interviews with a different sample of children aged 8–15, documenting their experiences during lockdown. The researchers contextualised the two datasets by drawing on international statistics, policy documents, data from public opinion surveys and public discourse. Data concerning Latvia and Lithuania provided a backcloth for situating developments in Estonia.

First, the article provides a brief overview of literature about the impacts of the pandemic on young people’s lives. Then follows a description of the methodology for the empirical work and a presentation of the findings. The discussion and conclusions indicate how the research was used to inform policy development.

## TRACKING THE IMPACT OF COVID-19 ON YOUNG PEOPLE’S Lives

Young people interact in different life domains, including family, friendship groups, school, community and via technology, all of which were affected by and had an impact on well-being during the COVID-19 crisis. Social and human science researchers around the world have explored the effects of the COVID-19 pandemic and ensuing lockdowns on young people’s lives. In the United States, for example, based on the reports of adolescents concerning their experiences of the pandemic, a qualitative study by psychologists (Scott et al., 2021) identified 14 thematic fields where the pandemic had affected everyday life. They included mental and physical health, family relations, friendships, social connections and community, attendance at important events, socio-economic performance, routines, observance of COVID rules, exposure to COVID risks, and the adoption of technologies. Psychologists in Spain (Idoiaga et al., 2020) explored children’s social and emotional representations of the pandemic and analysed how children coped during the crisis.

Studies in child psychiatry, psychology and public health in different parts of the world have shown that the pandemic increased child vulnerability and endangered mental health, resulting in a decline in subjective well-being (Fegert et al., 2020; Gadermann et al., 2021; O’Sullivan et al., 2021). In a wide-ranging review article, Chaturvedi and Pasipanodya (2021) highlight disruptions to school and social life as the main factors determining child vulnerability. Other researchers identify COVID-19 related stressors, such as high levels of anxiety and depressive symptoms, associated with perceptions of parental stress within families, causing new problems in parenting that influenced children’s ability to cope with the pandemic (Brown et al., 2020; Spinelli et al., 2020).

The COVID-19 pandemic caused momentous changes in patterns of interaction, due to the implementation of lockdowns and policies on social distancing in children’s lives. Family sociologists in Poland (Markowska-Manista and Zakrzewska-Olędzka, 2020) studied changes in family life caused by restrictions and limitations on mobility and social relationships. Comparative evidence from Switzerland, Canada and Estonia showed how the pandemic exacerbated inequalities between children across different life domains, especially within families, at school, with friends and in access to public services (Stoecklin et al., 2021). While increasing the importance of virtual spaces, government measures in these countries were found to limit physical interaction with friends, teachers and relatives by closing access to schools, playgrounds, and recreational activities.

From a sociological perspective, children’s experiences can be best understood by setting them within what has been described as a “social ecological framework” (Bronfenbrenner, 1979; Bronfenbrenner and Morris, 2006; Sallis et al., 2008). Bronfenbrenner’s model provides an appropriate theoretical and methodological framework for the present study by capturing children’s experiences through the mutual interactions between a child, her or his immediate environmental settings, and the wider socio-cultural contexts in which she or he is embedded (Scott et al., 2021). This approach considers children as dynamic social actors and reliable sources of information about their own lives (Casas et al., 2013; Mason and Danby, 2011).

## Methodology

This section describes the data collection and analysis in the two distinct phases of the project covering the periods in 2020 and 2021 when schools were closed, many families were confined to their homes, and the participants were distance learning at home.

### Data Collection and Analysis

The data corpus for the research was compiled from two sources: children’s diaries during the first lockdown in spring 2020 and semi-structured interviews with children documenting young people’s assessments and reflections on their lives during the lockdown in spring 2021.

On 18 March 2021, the Estonian Literary Museum launched a public collection initiative of lockdown experiences in the form of diaries. Such an initiative is not unique and was replicated in several countries: for example, the Koronakevät initiative by the Finnish Literature Society, the Pandemic Diary Project in Latvia, and the Pandemic Diary Research Project in Poland. In Estonia, the call stemmed from a tradition of collecting life stories, starting in the late 1980s and early 1990s, that laid the foundation for the life-story archive of the Estonian Literary Museum, which today holds over 4,000 life narratives. The collection is viewed as a cultural resource of (national) memory and is archived for an indefinite period. The material was not collected with concrete research objectives in mind but has provided a basis for a wide range of research projects carried out by scholars with a background in literary/narrative studies, ethnology, folklore studies, and history. Preservation and access to the materials in the Cultural History Archive are governed by archival laws and regulations (see http://www.folklore.ee/era/eng/procedure.htm).

Schoolchildren of all ages were invited to keep a daily record of their lockdown experiences, paying attention to changes in everyday life, and in their relationships with family members and wider society. They were encouraged to write about their fears and hopes as well as commenting on how lockdown was being handled by the authorities, and the support, if any, offered by national and local governing bodies. The call did not specify a minimal number of entries, their length or the required duration. It was published on the homepage of the Estonian Literary Museum and also promoted through online, social and traditional media. Schools were asked to create student assignments based on the call requiring participants to keep a lockdown diary or to write a memoir.

The data corpus for spring 2020 included 19 diaries by children aged 12–13 (grade six; 10 girls and 9 boys), and 20 personal narratives by upper secondary school students aged 17–18 (grade 12; 14 girls and 6 boys), that the students wrote as a school assignment. No personal data were collected about the diarists other than their grade at school, name and gender. The diaries covered the period from 18 March to 15 May 2020, with one diary entry per day. Some diaries included illustrations. The length of the entries varied, ranging from 50 to 200 words each. The diaries were handwritten in regular school notebooks. Personal records by older students were loosely based on the questions in the public call. They contained longer reflections (300–800 words) on the lockdown period and were submitted in digital form. With the consent of their parents, the children offered their diaries and reflections to the museum on a voluntary basis. For the purposes of the present article, the children’s identities were anonymised: only the first initials of their first names, sex and age group are indicated in the analysis.

The second data source contains transcripts of 24 semi-structured interviews, each lasting about 15–20 min, with children aged 8–15 (7 girls, 4 boys, 13 gender not specified), carried out as a practical fieldwork assignment for students attending a course on Children and Childhoods (3 ECTS) at the University of Tartu. The interviews document experiences of lockdown in spring 2021. The students were allowed to apply a convenience sampling method to find interviewees among children whom they knew, combined with an element of purposive sampling. Alongside the objective of carrying out a training exercise, the intention was to analyse the experiences of schoolchildren from different age groups, loosely defined. The purposive sampling method had the advantage of providing a mosaic of young people’s reactions to the pandemic across a wide age span, while contributing to the overall objectives of the training exercise. It had the disadvantage of not producing a representative sample of specific age groups as would have been possible in a larger-scale more quantitative study using a more rigorous sampling method.

The fieldwork process was supervised by the first author of the article in her capacity as course director. The interviews were carried out online or by telephone. A few interviews took place face-to-face observing social distancing rules. The interview frame was very simple: the children were asked what had changed in their lives compared to the situation pre-lockdown, what had improved or got worse. Children’s names and other personal data were not used. The children participated in the interviews on a voluntary basis and with parental informed consent: the interviewers approached the parents by telephone or by e-mail.

The authors were granted access to the handwritten and digital diaries. The interviews were first transcribed, and the interviewers wrote short fieldwork reports as part of their course assignment. The authors undertook in-depth analyses of the interview transcripts and diaries using a directed content analysis method consistent with the Bronfenbrenner’s social ecological framework (Hsieh and Shannon, 2005). The two datasets covered different phases in the pandemic. They were not directly comparable since the diaries were child-driven, and the interviews were led by adults. The combined evidence from the two data sources do, however, provide valuable insights into the cumulative effects of the pandemic, as it progressed, on family life in Estonia as experienced by children.

## ANALYSIS OF CHILDREN’S DIARIES: SPRING 2020

Before the outbreak in March 2020, COVID-19 was perceived by the public as something happening elsewhere in the world, mostly in Asia, that would never reach Estonia, as was the case with the H1N1 influenza virus in 2009. Since the introduction of lockdown on 12 March 2020, the rate of new infections had remained relatively low, around 20 for 100,000 inhabitants. As in the rest of the world, Estonian society was, nonetheless, exposed to an unprecedented situation generating the feeling of loss of control over everyday life, and causing anxiety and insecurity. Lockdown altered lifestyles and disrupted the enjoyment of living in a society that had become used to a high level of social protection and general well-being. For families, lockdown meant that many parents were unable to go to work due to temporary closure of their workplaces. Unless they were able to switch to teleworking, in most cases they were entitled to compensation from the government for loss of income. School closures resulted in children moving overnight to distance learning.

In Estonia, households with children form one fourth of all households and close to one fifth of children living with a single parent (Statistics Estonia, 2021). The majority of children living with a single parent maintain some contact with their other parent. Many children have visiting orders that had been agreed by the parents, a child protection specialist or court, which had to be followed by the child. The strong recommendation to stay at home served as a pretext not to follow the visiting order. In addition to the general disruption to routines that this caused, some children had to stay longer than they would have wished with one of their parents. Although data were not collected about individual living arrangements, it was clear from some of the comments in the diaries that different family constellations and socio-economic circumstances influenced how children coped with the impact of lockdown.

### The Impact of the Pandemic on Family Practices

Analysis of the diary entries suggested that the pandemic had diverse effects on children’s subjective well-being and development. Many homes turned into over-populated multifunctional places 24/7, with both positive and negative impacts on relationships. The children’s diaries indicate that they fully understood the seriousness of the crisis. Several diaries contain statistics about the spread of the virus in Estonia and elsewhere in the world. Boys more often than girls documented COVID-19 statistics in their diaries. Girls focused rather on describing relationships and activities with family members and friends. One boy’s report showed awareness of the pandemic crossing country borders and continents on a daily basis:

There are more than 200 cases of corona in Estonia so far and it is quite horrifying since the number has doubled since yesterday (16 March). Today there was the first corona death in Estonia (25 March). Today there was a new record in Italy but this is very sad record; 1,000 people died during the last 24 hours (27 March). Corona has taken over almost the whole world by now except a few countries in Africa (30 March). (Ka, boy, grade 6)

The diaries of both younger (grade 6) and older (grade 12) students recorded the responses of their family members to information about the spread of the virus, demonstrating feelings of fear and insecurity. One upper school student was concerned about the health and well-being of his father who had to commute between different countries during lockdown. He wrote: “That night I got very little sleep because my father told me that his … sleep routine was all messed up.” (Ma, boy, grade 12) For him, an additional source of tension was the fear that the father could transmit the virus. He was upset by his mother’s perception of the father as a potential source of health risk to the family and the self-isolation measures that she thought necessary. Other boys expressed concerns about transmission of the disease:

The elderly care home is in quarantine … it is quite scary that it is so close. We don’t know if it is only life threatening for older people or whether younger people also die. (Ol, boy, grade 6)

The diaries and personal reflections commented on different safety measures that the children and their families followed. They expressed concern about people who did not follow the rules. The diary entries suggest that the public message requiring everyone to help keep the virus under control by following the rules was taken seriously by both the children and their families. One student wrote that he wished:

…people would stay at home, because this is in the best interests of their health and that of the others. The longer we put up with staying at home, the faster we get out again. (Sv, boy, grade 12)

Children of all ages expressed a positive attitude and understanding as to why lockdown and stringent restrictions were necessary. Children described, for example, how they followed the rule about social distancing:

With my friend we wanted to go to Lossimäed [a nice park] but there were people there. We waited for them to leave but they did not, and so we ended up not going at all. (Ro, boy, grade 6)

A male upper school student commented on changes in the public health instructions, stating that: “Before leaving home we need to clean our hands and wash them right away after returning home.” (Sv, boy, grade 12) He and several other upper school students mentioned postponing or cancelling visits to older family members. One girl, whose grandmother lived with the family, and who used to have friends come to her home, wrote: “My mother has established a rule that no one can visit us right now. I respect my mother’s rule and my grandmother’s health.” (Gr, girl, grade 12) Another upper school diarist commented:

It is really sad to witness that there are still people who throw parties or ignore the restrictions.… We all need to do what we are asked to do and follow all instructions, then we can all successfully manage this situation together. (An, girl, grade 12)

Older children analysed the role of different media in spreading information about the virus and government measures:

When the situation became more serious, global panic started … that was, of course, spread by social media … At times of crisis like that, leaders and governments can show how weak or strong they are. (Ti, boy, grade 12)

Some older students expressed frustration with news that focused only on negative events, resulting in them giving up following the news altogether (Ka, girl, grade 12). However, most students in the older age range did not report perceiving very high risks to their own safety or those of their family members. One girl reported that, although the number of infected people had risen to 679, with 3 corona deaths:

I know that there are few corona deaths in Estonia. My strong immune system would cope well with the virus. In my family, people are protected and the possibility of them getting the virus is small. (Ma, girl, grade 12)

In the older age group, children who stayed at a school dormitory during term time enjoyed being at home with family members, doing gardening and maintenance work around the house, and helping younger siblings to manage their schoolwork. While younger students frequently mentioned new family activities such as going for a jog together, doing puzzles or trying out new recipes, they occasionally became frustrated with their siblings and conflicts developed. About a third of the children reported an increase in anxiety and tension in relationships at home. One girl in grade 6 wrote:

We fought with my brother against our sister for half an hour or so. I got some bruises. But in the end, everything worked out [and] we made up. (Kr, girl, grade 6)

Younger children often developed conflicts with their siblings; they were usually solved very quickly, and normal friendly socialising continued. A girl whose father was commuting to work wrote: “My father has worked in Virumaa [North-East Estonia] the whole 3 weeks. I cannot stand my mother and brother alone anymore. Hell.” (An, girl, grade 12) Some children also mentioned problems with their parents:

I was too tired to get up right away. When I was getting dressed, my stepfather shouted that I need to feed my cat right away or he will take it to the shelter. (Be, girl, grade 6)

Children in the younger age group whose parents were separated wrote about missing the parent with whom they did not live on a daily basis: “I’d like to spend the last week of school with my real father.” (Sa, boy, grade 6) Some of the younger boys also mentioned suffering from being separated from their grandparents: “Grandparents wanted to visit us, but our parents did not allow that, it was such a pity.” (Ol, boy, grade 6).

Lockdown was generally associated with greater stability and more opportunities to share leisure activities or do housework and small household repairs together, thereby generating greater closeness and more dialogue and new routines within families. Children described growing closer to their families but also suffering from having to live in such close proximity all the time, especially with younger siblings. Some children also wrote about tensions and feelings of isolation in the home, not being able to share personal matters in a trusting atmosphere, being pressed to do too many home chores on top of managing schoolwork, and missing out on parental attention. They regretted not being able to see close relatives, including grandparents, parents who were living apart from them or who had to travel to work, and were concerned about their welfare.

### The Impact of the Pandemic on School Life and Social Contacts

Schools in Estonia had already been using web applications such as eKool and Stuudium to facilitate teaching and learning several years before the pandemic, and collections of online study materials were freely accessible for all students, for example Opiq. These tools were used for assigning tasks, feedback and grading, monitoring progress, communicating with students and parents, posting study materials and submitting assignments. Nevertheless, neither children and parents nor teachers had a clear vision of how full-time e-learning would work. Children’s experience of distance learning varied. New learning environments and requirements challenged children who previously had learning difficulties, those with special educational needs and those where internet access was limited. The pandemic erased the distinction between school time and free time, cancelling extracurricular hobbies or moving them online, thereby considerably constraining the range of free-time activities. Although few children felt they could not handle e-learning at all, relatively few did not experience some problems with distance schooling. Moving schools to distance learning was the protective measure that the children raised as posing the greatest challenges for them.

Young people in both age groups commented in their diaries on positive aspects such as being able to sleep longer and having greater freedom in managing their time. Upper school students, in particular, appreciated being in charge of their time and able to study independently. One girl pointed out that she no longer felt exhausted at the end of the school day since she “managed to plan her school day more productively than when she was at school”, and she was able to “think independently” (Vi, girl, grade 12).

In the younger age group, boys reported more positive attitudes to distance learning: “Most of us are glad about that, me included. You can sleep longer in the morning and study whenever you want.” (Ka, boy, grade 6) Another boy noted that distance learning seemed easier: “I like … to study in my own room. I can also take whatever I want to eat from the kitchen.” (Ol, boy, grade 6) Students in different age groups mentioned as a positive aspect of staying at home more varied and better tasting meals, indicating that families were finding time to support their children during lockdown.

Children in both age groups found it difficult, and physically straining, to sit at the computer all day. They complained about problems with self-motivation, considered school assignments monotonous and missed the contrast between time spent in and outside school:

Why do we have so much homework? Where has my motivation gone? As I could not sleep at night, I take a nap, then start doing schoolwork at about 4 pm and end at 2 in the morning. (Ja, girl, grade 12)

Some of the diarists mentioned experiencing mental health problems, the failure of parents to understand their problems, and the need to seek online professional psychological counselling.

Several younger students expressed their frustration with distance learning. A male diarist complained: “I am so tired of distance learning, since I couldn’t get out of the house and meet up with my friends. This routine is getting on my nerves.” (Ma, boy, grade 6) Particularly in the younger age group, sleep problems were quite frequent and could be viewed as a major effect of the pandemic and the change of routine, as admitted by one girl:

Though studying is necessary, studying during lockdown upsets me and even drives me crazy. I woke up at six o’clock again. I contemplated starting to get going with my schoolwork, but nevertheless fell asleep again ... [At night] our parents asked us to put the lights off and the laptop away and go to sleep. We put the laptop away and the lights off but did not go to sleep. (Kr, girl, grade 6)

Some younger students wrote about skipping a whole night’s sleep or even reported not sleeping for several nights in a row. For upper school students, not knowing whether they would be able to graduate with good results and continue their education was the greatest source of distress, far exceeding fear of the virus. By contrast, some younger students noted that the less stringent demands being made on them by distance learning made it more tolerable:

I even like [distance learning] now, because, firstly, I can sleep longer and, secondly, I don’t need to take everything so seriously and, thirdly, in some subjects we...simply get pass or fail. (Ka, boy, grade 6)

A sociological study among teachers, parents and children in Estonia in 2020 revealed that about one third of children were satisfied with distance learning, another third had difficulties in adapting to it, and the rest said that it made no difference for them (Lauristin et al., 2020). Distance learning challenged children’s self-regulation skills. Some children enjoyed it because of the relative independence to introduce their own daily time schedules and distinguish between school, hobbies and leisure. But other children missed the pre-pandemic ways of teaching and learning and were not able to adapt to new forms of schooling without the atmosphere in the classroom and opportunities for open and direct discussions, questions and answers.

Children in both age groups commented that social distancing from friends was the most difficult challenge they faced. Many children, especially in the younger age group, wrote about missing friends and opportunities to hang out with them:

I miss my friends; it seems I haven’t seen them for years. I miss school. I wish the virus would go away so that regular routine can continue. (Ca, girl, grade 6)

I would so much like to go back to school as I have nothing to do at home. (Ro, boy, grade 6)

I miss school, first of all, all my friends. Also, schoolwork progresses much faster at school. (Ol, boy, grade 6)

Younger children also missed teachers and the school atmosphere for which online classes were no substitute. Although younger students rarely recorded difficulties in managing schoolwork, they clearly preferred face-to-face learning.

The reflections of older students showed that friends played a central role in handling problems and maintaining a positive outlook on life. One diarist, who was suffering from forced separation from her boyfriend and problems with managing schoolwork, mentioned the communication channels she and her friends adopted, while admitting that this format of communication was not an adequate substitute for those used in pre-pandemic times. In her life, problems piled up, but her mother was aware of them and was supportive:

Life within these walls in these conditions without good friends is nothing short of a catastrophe. At least [friends] check up on me *via* phone and messages. [When my] Mom … sees that I’m messed up, she gives me a break with schoolwork and chores at home. (An, girl, grade 12)

Girls and boys, particularly in the younger age group, described taking up new hobbies or rediscovering old ones, as well as finding ideas for activities on the internet: “In the evening I watched different YouTube videos on things to do during lockdown and got a lot of new ideas.” (Me, girl, grade 6) Other diarists found photography tips (Kr, girl, grade 6), instructions for making wrist bands (La, girl, grade 6), for playing the piano and composing (Ma, boy, grade 6), or they used the time at home to pursue other artistic activities: “I have had more time to practice my guitar and … out of utter boredom I even messed around with watercolours a bit!” (An, girl, grade 12).

The diaries demonstrated that young people reacted and adapted to the challenges they perceived in their everyday lives in many different ways. In the majority of cases, they displayed resourcefulness, flexibility and effective coping strategies. They appreciated the support they were given at home and by their schools. Yet, the diaries revealed a number of problems, the most important for both age groups being the lack of direct contact and face-to-face socialising with friends, teachers and schoolmates.

## ANALYSIS OF THE INTERVIEWS WITH CHILDREN: SPRING 2021

Although COVID-19 death rates remained relatively low by international standards, a new lockdown was imposed on Estonian society between March and June 2021 in response to rising rates of infection (Our World in Data, 2021). Schools reverted to distance learning, shopping centres, theatres, cinemas and all places of entertainment were closed, wearing masks was compulsory in public places, the 2 + 2 rule was reintroduced. Local governments decided to distribute food packages through schools to compensate for the loss of free lunches, previously offered at school to all students. Compared to the first lockdown in Spring 2020, the government tried to keep the society as open as possible. As a result, the lockdown introduced on 11 March 2021 was criticised for coming too late to prevent the peak for new cases being reached on the 17 March 2021. The government’s priority was to avoid the collapse of the healthcare system. In public discourse, young people were blamed for transmitting the virus while being unprotected against it because they had not been vaccinated. The economic coping capacities of families were endangered because central government had decided to end the income-loss compensation scheme despite rising unemployment in the second half of the lockdown. After a year living with the pandemic, the level of anxiety experienced during the first wave had lessened, but weariness and discontent with the regulations were contributing to emerging mental health problems among young children as reported in the interviews.

### The Impact of the Pandemic on Family Practices

The interviews with children showed that their attitudes towards the virus and their patterns of behaviour were largely determined by the way their families handled the pandemic. As in the diaries, in the interviews children recalled that they had felt frightened and insecure a year earlier in spring 2020. They told how they had followed the very strict self-isolation measures, for example by staying indoors for months, and/or avoiding all public places. At the time of the interviews in April 2021, the whole society was better prepared for living with the virus than they had been during the first wave of the pandemic. Children felt safer and expressed the belief that the danger could be minimised if all the safety measures were followed. They considered themselves to be well informed about the risks and safety measures.

The interviews confirmed that most of the children were continuing to follow safety regulations. One child recounted: “When my older brother comes to visit us from Tallinn where he goes to school, he wears a mask at home.” (boy, aged 9) A few parents or family members did not consider it important to follow any safety measures or even acknowledge the existence of the pandemic: “I don’t care one bit about the virus, my father said that there is no such thing as the virus.” (boy, aged 14) In the interview, he made clear that, in his everyday life, he followed safety measures loosely; for example, he talked about meeting up with friends on a regular basis, whereas most other interviewees noted that they had few opportunities to see their friends and were suffering from the lack of regular contacts, because they considered it important to follow the safety precautions.

Compared to the accounts in the diaries, the interviewees more often spoke about weariness and strain in family relationships. They complained about having to spend time with their family members 24/7, resulting in tensions and arguments and occasional conflicts with younger siblings. But most children were content with their lives, reflecting their subjective assessment of how they were adapting to the new reality. Many children described being able to manage schoolwork and felt they had the necessary support. In cases where significant changes had taken place in their parents’ lives, due to the considerable reduction in income, loss of employment, or fatigue and frustration resulting from the additional responsibilities at work and at home, children did not necessarily observe a worsening of their home environments. In some instances, they talked about noticing the greater workload on their parents, but were supportive and appreciative of the way their parents were coping. Where the family’s income made it possible to procure extra services, the children rated their quality of life highly. For example, one family had hired a private teacher who came to their house and helped the family’s two children with schoolwork 3 days a week.

As in the diaries from spring 2020, the children mentioned family activities − going for walks and outings and cooking together − as well as new activities that helped them to endure lockdown. They also remarked on holidays and birthday celebrations being scaled down, admitting that they preferred bigger gatherings and outings on festive occasions.

### The Impact of the Pandemic on School Life and Social Contacts

When asked about their recent experiences during lockdown in April 2021, the children described the pandemic as having lasted one and a half years. For adults, the period under consideration at that time was perceived as having lasted for a calendar year; for children, it represented three school terms that had been lost because they had been studying at home or moving in and out of lockdown, making them miss contact hours at school. They therefore perceived time spent remotely as lasting longer than the time spent at school.

The schooling situation differed from that in spring 2020 since students, teachers and parents had, meanwhile, developed skills in handling distance teaching and learning. Schools were also technically better equipped, and teachers had adopted more effective online teaching techniques. During distance learning periods, the number of videoed classes increased considerably, often equalling the number of regular classes. The interviews with children showed that, in general, they believed distance learning was progressing smoothly. Most children described managing distance learning relatively well and even expressed the hope that, in the future, distance learning (e-learning) would be integrated more thoroughly into daily learning schedules. As noted in the diaries, the interviewees valued the greater degree of independence and self-management opportunities made possible by distance learning.

Yet, some children felt the need for more support from individual teachers if they perceived that they were lagging behind with schoolwork and lacked help to get back on track. The interviews showed that children of primary and lower school age (7–15) were often relying heavily on their parents’ support and/or the support of older siblings, sometimes to a greater extent than on help from school. These children pointed out that schoolwork had become more mechanical, and that they did not have enough time to focus properly. They admitted that they often cheated, for example by handing in school tasks that someone else had completed. The children were, consequently, worried about their results.

The most widely reported and strongly felt impact of the pandemic was on social contacts. This effect had clearly worsened considerably since spring 2020. Most children said that they felt estranged from their friends and admitted that virtual communication did not make up for regular face-to-face contact. Some children said that they had lost all their friends and were now completely alone. Children also observed changes in the behaviour of their friends and classmates. They noticed other children around them becoming more nervous, getting frustrated and more easily upset. In some cases, children detected mental health problems among their friends, including the infliction of self-harm (cutting). Children who had been active in playing sports (group gymnastics and basketball), or some other hobby that could not take place online, suffered from the cancellation of these activities and the associated loss of opportunities to socialise, including participating in competitions. Almost all the interviewees felt tired and bored. Many expressed fears that the world might never return to the way it was before the pandemic, and that they might never get back to their normal, regular lives.

## Discussion and Conclusions

An important aim of the empirical research described in this article was to understand how findings from a study of the impacts of the COVID-19 pandemic on children’s lives in Estonia might be used to track the interactive process between policy as promulgated in lockdown measures and as reported in the behaviour of young people. Published reports, opinion polls and grey literature provided background evidence about the wider socio-political factors shaping government responses to the health crisis and, in turn, influencing young people’s perception of the effects of lockdown on their lives.

The impact of school and workplace closures, combined with measures restricting mobility, social and physical contact, on the socio-psychological climate of families with children was immediate and profound. It transformed the reality of everyday life. In all the Baltic States, the workload for parents assisting children with online learning and taking care of pre-school children increased markedly. In addition, parents who commuted across borders for work in the Baltic States were hit by restrictions on border crossings and by border closures.

A national study of students, teachers and parents in Estonia during the first lockdown in 2020 indicated that, depending on the type of work, 36% of parents were using their homes as offices, and 44% reported an accumulation of responsibilities and increased stress levels, especially for parents with small children and families with several children (Lauristin et al., 2020). Lithuania registered more than a doubling of the level of anxiety and a marked deterioration in the emotional climate during the 2020 lockdown (Eriksonas, 2020).

Income inequality between families increased markedly during the pandemic: in late May 2020, 55% of the population reported a decrease in their own or their family’s income level, and 36% of the unemployed said they had lost their job during the crisis (Riigikantselei, 2020). A year later, when unemployment support schemes were withdrawn, 25% of the population reported problems with coping financially (Riigikantselei, 2021). Young people’s diary entries during the first wave of the pandemic in 2020 suggested that a positive family environment could alleviate the negative effects of the lockdown and help them to cope with unexpected changes in their everyday lives. Stable family life encouraged communal activities, such as table talk, board games and outdoor activities as a family group, although the children were already expressing concern about the effects of lockdown measures on their interaction with friends and the wider community. The restrictive measures in spring 2020 had been introduced so rapidly that it was not possible to capture their impact, making it difficult to predict how they would be perceived by children. The voices of parents and teachers became increasingly audible in policies and public debates presenting the problems of coping with distance learning and other impacts of the pandemic on everyday lives.

Analysis of the interviews in spring 2021 suggested that, over time, working from home and living in close proximity increased boredom and strain in family relationships, impacting negatively on subjective well-being. The children were particularly affected by the loss of physical and social space due to confinement at home and the limits imposed on mobility and social interactions, even if these conditions were partially mitigated, at least initially, for children because they were able to spend more time with family members.

Problems of domestic violence were not mentioned in the diaries or interviews, although they were being widely discussed in public debate in early 2021. Social workers and psychologists acknowledged that, during lockdown when family members were living in close proximity, such cases had become more prevalent, but that the victims seemed to be too frightened to report abuse. One psychologist described how the presence of other family members threatened children’s privacy and made it difficult for them to speak about personal experiences of abusive behaviour (Haldre, 2020).

The restrictive measures posing the greatest challenges to children according to the accounts in the diaries and interviews resulted from having to reorganise their studies and to forego most of their regular socialising activities. Both in spring 2020 and 2021, distance learning was introduced universally, with teachers providing daily schedules of classes to enable children to structure their day. By spring 2021, the capacity of schools to carry out distance learning had increased considerably. On both occasions, densely populated areas with high infection rates were treated similarly to sparsely populated rural areas with low rates. The adverse effects of distance learning were greatest for children who were most dependent on direct communication with the teacher in a real classroom for motivation and encouragement whether they lived in an urban or rural setting.

In line with the findings from the diaries and interviews, educationalists became increasingly concerned about the loss of learning, particularly for children in the most deprived families. In spring 2021, ideas were being sought to enable children to catch up at the end of the school year. A proposal that found fruitful political soil and public support was the organisation of “summer learning camps” for children at an educational disadvantage as a result of the pandemic (Otsmaa, 2021). On 27 May 2021, the Minister for Education and Research announced its decision to support student camps aimed at stimulating interest in learning and “re-socialising” in preparation for the return to school. Funding was also made available for schools to provide additional support for pupils and for the purchase of laptops to loan to students, and for improving internet access, speed and reliability.

Following a suggestion from the Scientific Advisory Board in May 2021, the Minister for Education and Research (2021) stated in an interview that grades one to six and upper school classes should, in future, be closed only during extreme circumstances. It seemed likely that the combination of distance and in-person learning would find support across the educational landscape and that formal and non-formal education would become more closely integrated (Estonia Education Forum, 2021). The analysis of children’s perspectives on distance learning in the two phases of the research demonstrated that online communication did not compensate for real-life classes, although the children in the study were not averse to the adoption of blended solutions.

A study carried out in spring 2020 had revealed that 10–15% of children were likely to be severely disadvantaged by distance learning (Lauristin et al., 2020). The diary entries provided evidence of how the socio-economic and cultural inequalities between children in their everyday lives had been exacerbated by government policies. The interviews indicated that the lockdown measures presented significantly greater risks and challenges for the mental health of families and children during the second wave of the pandemic in 2021, even though they were less stringent.

The inclusion in the study of two different age groups demonstrated that the main challenges arising from the lockdown measures—limited social contacts and distance learning—were shared by both age groups but with different effects. Children in the younger age group saw socialising as an important part of schooling, whereas the main concern for the older children was the insecurity of their prospects for continuing their education. Markers of mental health issues were present in both age groups, but only children in the older age group were aware of the problem and ready to seek professional help.

Analysis of the diary entries had shown that some children recognised that they were at an advantage because their homes were well equipped with technological resources, and their parent(s) possessed the knowledge and skills needed to assist in schoolwork and were able to spend more time on family activities than before the pandemic. These children felt safe and protected, even though they missed direct social contacts with friends. The interviews, and to some extent the diaries, showed that the children who did not share these advantages experienced difficulty with self-motivation, failing friendships and deteriorating mental health. They were most likely to be suffering from the lack of parental support with their schoolwork and face-to-face socialising with friends, to feel isolated and excluded from the wider community. When they encountered problems with schoolwork, they felt that they would have benefitted greatly from more direct contact with teachers.

An Estonia Education Forum (2021) confirmed that these disadvantaged children needed direct interaction with teachers and other children in the classroom to advance their educational outcomes, as well as their social skills. The impact of social distancing on children’s mental health served as a warning sign. To deal with the deteriorating mental health situation in spring 2021, at the time when the interviews were being carried out, psychologists and therapists launched online counselling sessions and public campaigns to draw attention to strategies for coping with mental health problems resulting from social distancing. Compared to the lockdown in 2020, children gained more visibility in policies during the lockdown in 2021 mediated by educationalists and psychologists.

Although, compared to other countries, national surveys found Estonian adults to be more satisfied with the decisions taken by the government (Ahrendt et al., 2021; Riigikantselei, 2021), our findings showed that the children were critical of government responses, in particular rules on social distancing. However, they also understood the need for them. Evidence from the diaries and interviews reported in this article suggests that political decisions affecting the delivery of, and access to, any of the services provided by local authorities could have severe implications for children both by exacerbating the negative impacts of the pandemic on their well-being and by increasing the social inequalities between them. The pandemic impinged on the wider socio-economic and cultural contexts in which children and their families are embedded. Although the decrease in GDP was relatively small in Estonia, confirming the flexibility of a small economy, as the crisis was prolonged, the increase in national debt threw into sharp relief the sustainability of public services and the risk of austerity. The increase in the education budget was designed to mitigate the negative impact of the pandemic on children’s education.

The diaries and interviews confirmed the value of Bronfenbrenner’s social ecological model (Bronfenbrenner, 1979) in helping to understand children’s experiences during the pandemic within the multiple interactive contexts of family, school and the wider community. They demonstrated bi-directional influences between child and context. At the micro-level, the pandemic challenged the children’s capacities of self-regulation and self-confidence, endangering their identity and self-worth; it also modified their interactions with other family members by creating the risk of isolation while giving a new impetus to mutual relationships.

Major modifications in the interactions between children and the wider community occurred in the educational environment, where a direct social constructionist learning process in a shared reality was replaced with online distance learning. Not only the study process but also children’s relationships went online, thereby totally modifying and transforming the process into “friendships from a distance”, causing major protests among children. Time spent on the web was modified and expanded to enable greater access to technology-supported interactive spaces. The children accused the pandemic regulations of taking away their autonomy and freedom. Some direct forms of interaction—maintaining friendships and effective learning—became indirect, mediated by the internet, while some previously indirect interactions mediated by family units within the wider community—managing the health crisis and government restrictions—became everyday practices for children.

This study has analysed the impact of national lockdown measures on children’s familial, educational and social lives in Estonia. The accounts of young people’s experiences of the pandemic showed that they were aware of the need for protective measures and prepared to comply with them. The analysis in the article has also considered the role played by policies introduced by central government to mitigate and reverse the short- and longer-term effects of the restrictive measures on children’s well-being. The adequacy of these policies remains to be seen. More could be done, for example, to improve the targeting of support measures for children, including access to mental health counselling. Whatever the outcome in the longer term, the findings from the study confirm the value of listening to young people and of taking their experiences seriously throughout the interactive policymaking process.

## Data Availability

The raw data supporting the conclusions of this article will be made available by the authors, without undue reservation.
